# A taxonomic-based joint species distribution model for presence-only data

**DOI:** 10.1098/rsif.2021.0681

**Published:** 2022-02-23

**Authors:** Juan M. Escamilla Molgora, Luigi Sedda, Peter J. Diggle, Peter M. Atkinson

**Affiliations:** ^1^ Lancaster Environment Centre,; ^2^ Centre for Health Informatics, Computing and Statistics (CHICAS), Lancaster Medical School, Faculty of Health and Medicine, and; ^3^ Lancaster Medical School, Faculty of Health and Medicine

**Keywords:** species distribution models, presence-only data, tree of life, multivariate conditional autorregresive models

## Abstract

Species distribution models (SDMs) are an important class of model for mapping taxa spatially and are a key tool for tackling biodiversity loss. However, most common SDMs depend on presence–absence data and, despite the accumulation and exponential growth of biological occurrence data across the globe, the available data are predominantly presence-only (i.e. they lack real absences). Although presence-only SDMs do exist, they inevitably require assumptions about absences of the considered taxa and they are specified mostly for single species and, thus, do not exploit fully the information in related taxa. This greatly limits the utility of global biodiversity databases such as GBIF. Here, we present a Bayesian-based SDM for multiple species that operates directly on presence-only data by exploiting the joint distribution between the multiple ecological processes and, crucially, identifies the sampling effort per taxa which allows inference on absences. The model was applied to two case studies. One, focusing on taxonomically diverse taxa over central Mexico and another focusing on the monophyletic family Cactacea over continental Mexico. In both cases, the model was able to identify the ecological and sampling effort processes for each taxon using only the presence observations, environmental and anthropological data.

## 1. Introduction

Estimating the geographical distribution of species, conditioned to their ecological niche, is crucial for assessing the risk of species extinctions, habitat restoration and forecasting the effects of climate change on biodiversity [1,2]. Species distribution models (SDMs) are designed for these purposes and have become essential tools for diagnosis, decision and policymaking at regional and global scales [3]. SDMs are effective in characterizing the natural distributions of species when the sampling observations are properly designed to fit the model’s assumptions [4,5]. These assumptions typically are: (i) the probability for a target species (*P*) to occupy a given area is independent from other species [6,7] and (ii) *P* is at *equilibrium* with its environment. That is, species are present across all environmentally suitable areas and are absent in unsuitable environments [8]. While the value of SDMs in decision-making and environmental assessments is indisputable, the aforementioned assumptions greatly limit their scope in application.

Research in community ecology has long recognized the interplay between environmental niche, species interactions and stochastic variability across space in determining distributions and species dynamics within ecological communities [9]. Omitting species interactions in SDMs (as when assuming independence between species) could lead to misrepresentation of species distributions, as some species are more likely to occur under the presence of others. Joint SDMs (JSDMs) are designed to address this problem by jointly modelling multiple species simultaneously. JSDMs provide more flexibility for assembling multiple species, resulting in models with greater support in ecological theory, inferential and predictive power [10]. For example, JSDMs have been shown to be effective in modelling rare species. This is achieved by allowing observations of conspicuous species to inform the likelihood of the rarest ones [11,12]. In particular, Bayesian-based hierarchical models have been demonstrated to be effective in modelling uncertainties for a wide range of JSDMs [10,13] by specifying random effects using different hierarchical levels to capture correlations between distinct taxa and ecological relationships. For example, Aderhold *et al.* [14] proposed a model for reconstructing species interaction networks using Bayesian change-point frameworks, and Jamil *et al.* [15] used generalized linear mixed models (GLMM) to incorporate the effect of species traits in response to the environment and other species occurrences.

Accounting for the simultaneous effect of multiple species is computationally expensive. In recent times, this limitation has been circumvented by advances in high-performance computing and computational statistics, facilitating the inference of complex statistical models using Markov chain Monte Carlo (MCMC) and, consequently, the development of more complex and statistically sound JSDMs. Early approaches for JSDM include: a hierarchical approach for binary responses (presence–absence) [16] and a geostatistical model for co-regionalization [17] to model a spatial effect per species. Later, Clark *et al.* [18] proposed a hierarchical model for abundances and presence–absence for multispecies using a zero-inflated Poisson process to account for the bias in the number of zeros related to abundance data. In recent years, approaches by Thorson *et al.* [19] and Ovaskainen *et al.* [20] used latent factors to model the whole community level with a single spatial covariance function for each latent factor. Later, Ovaskainen *et al.* [21] proposed a framework that uses phylogenetic information to reduce the number of parameters and gives a comprehensive baseline for characterizing species assemblages through hierarchical models. Their research was extended recently in Tikhonov *et al.* [22] with the inclusion of spatial latent factors (using Gaussian predictive processes). The mentioned approaches provide deeper understanding of ecological processes at different scales (i.e. from community to species) by relaxing the assumption of independence between species. This generalization of the model assumptions gives more accurate representations of the species distributions. Nevertheless, frameworks of this kind keep the assumption of environmental equilibrium and, therefore, rely on presence–absence observations.

The assumption of environmental equilibrium is usually problematic in practice, especially in analyses that cover large geographical areas. While presence–absence SDMs are statistically sound and fully characterize the probability of presences, the input data require a careful sampling design, specific to the research question. Another consequence of this assumption is the need for accurate data on absences. Failure to do so may lead to invalid conclusions, as the modelling assumption will no longer be valid. Obtaining records of absences that truly represent unsuitable environments is typically difficult and expensive, as they require intense sampling effort. As such, the availability of this datatype reduces their application to only a limited variety of studies.

An alternative to presence–absence SDMs are models that use presence-only observations. These types of models typically use data from opportunistic observations. Presence-only observations are easier to integrate across studies, at the cost of higher predictive errors, heterogeneous sampling designs, over-representation of taxonomic groups and bias across spatial and temporal dimensions, to name a few. See Dickinson *et al.* [23], Beck *et al.* [24], Isaac & Pocock [25], Franklin *et al.* [26] for more examples. Presence-only SDMs (PO-SDMs) need to account for the inherently biased nature of the input data. To do so, PO-SDMs require additional assumptions on how absences are conceived. These assumptions are necessary to fully specify an identifiable model and valid probability distributions [27,28]. As such, both types of models serve different purposes and complement each other to span a larger variety of applications. Specifically, PO-SDMs have made important contributions by facilitating the integration of data and allowing synthesis of ecological knowledge from regional to global scales (e.g. [29,30]). The appearance of global repositories of biodiversity data like the Global Biodiversity Information Facility (GBIF) [31], the eBird database for bird sightings [32], the PREDICTS global database on terrestrial biodiversity [33] and the Disease Vector Database [34] have opened the possibility to easily integrate the large amount of biodiversity data used in PO-SDMs. Despite this, to our knowledge, presence-only models for multiple species distributions have received relatively little attention, arguably due to demanding computational requirements. This is not the case, however, for single-species distribution models (SSDMs), where several modelling frameworks have been proposed within the last decade.

Single-species PO-SDMs aim to infer the relative probabilities of occurrences using external sources of informative data as a proxy for representing absences [4]. These models are referred in the literature as presence-background SDMs, with the maximum entropy (MaxEnt) algorithm [35,36] one of the most popular approaches. Phillips *et al.* [37] demonstrated that using informed background data (one with similar characteristics to the target data) significantly increases the model accuracy in terms of the relative probability of occupancy when observations from similar species (i.e. collected by similar methods) are used as background data (called *target group*). The joint modelling of both observations (i.e. target group and species of interest) accounted for the sampling bias, allowing the estimation of relative probability of occurrence given the target group. Parallel to the use of background information, the specification of spatial random effects for capturing the spatial variation across the study regions [4,38] provided an important improvement in SDMs, specifically the use of hierarchical models as a sound framework for inferring probabilities of binary variables (i.e. presence–absence). A relevant example is hierarchical presence–absence models with spatial random effects [39], implemented in the R package hSDM (hierarchical Bayesian SDMs) [40]. The combination of jointly modelling the sampling effort and the environmental niche, together with the specification of spatial random effects became, in recent years, a topic of ample research in SSDMs. Examples of this start with the early work of [41] accounting for a joint effect of the sampling bias and the ecological suitability (ES) of population counts. Along these lines, more complex models were proposed. See for example, Illian *et al.* [42] using marked point processes or Golding & Purse [43] using Gaussian processes. Recently, Gelfand & Shirota [44] proposed a hybrid approach using presence–absence data to inform partial realizations given by the presence-only data under a preferential sampling setting. These approaches have been shown to be effective in reducing the sampling bias. Nevertheless, they are designed for SSDMs, which assume no interactions between other species outside the environmental equilibrium. Neglecting these interactions could lead to inconsistent inferences of the species distribution, as pointed out by Clark *et al.* [18].

We consider that there is a knowledge gap in JSDMs for presence-only data. A proposal of this kind would help environmental and ecological researchers to infer and predict species distributions in a new variety of applications by integrating opportunistic observations from several sources. Here, we propose a hierarchical multilevel model for multiple species distributions using presence-only data. We propose a generalization of the classical modelling assumptions of SDMs by allowing the inference of species occurrence, conditioned to the presences of other species and using a presence-background framework for reducing the sampling bias of the occurrences. We use the taxonomic tree of the taxa of interest to obtain an *intrinsic* informative sample (i.e. independent from external information) to inform the likelihood of all taxa. The intrinsic informative sample serves as background information (in the sense of Ward *et al.* [28]) to define an identifiable JSDM based on presence-only data using the evolutionary (taxonomic) structure of the natural classification [45]. In contrast with traditional PO-SDMs, where the background sample is selected by the modeller given the characteristics of the studied taxa, our approach uses the available information to derive an informed background sample (hereafter called complementary sample) without the need for the modeller’s intervention. It is worth noting that the model’s specification and the algorithm for deriving complementary samples are independent and, therefore, it is possible for the modeller to use other sources for defining background data.

The paper is structured as follows. A description of the model is presented in §2. Section 3 tests the model performance on simulated data. The model was applied to two case studies, one located in central Mexico and using diverse taxonomic occurrences (§4) and, a second one, focusing on cactii across all Mexico (§4.2). Finally, §5 discusses the implementation, findings in the study cases, and future research, which builds on the fundamental approach introduced here.

## 2. Methods

**Figure 1.**
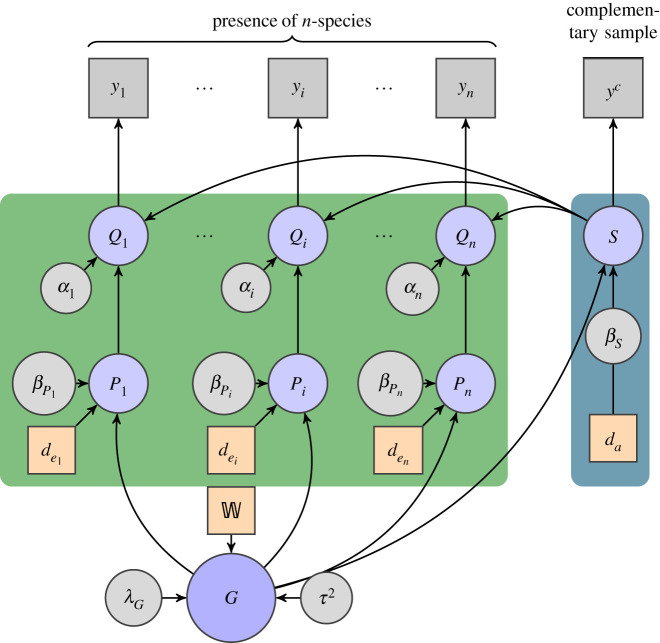
Directed acyclic graph representing the multi-species model. Nodes in squares indicate data. Grey squares are observed records and circular grey nodes represent the parameters used by the latent variables. *y*_*i*_ is the presence of species *i* and *y*^*c*^ is the complementary records of $\overset{n}{\underset{i=1}{⋃}}y_{i}$, i.e. the records that are not from species 1, … nor *n*, relative to the available dataset (Ω) or a subselection of it. Orange squares are covariates, $d_{e_{i}}$ for environmental based and *d*_*a*_ for anthropological based, associated with the sampling effort (dark blue block). Nodes in blue circles represent latent variables where: *Q*_*i*_ is the mix between the sampling effort *S* and the corresponding ecological suitability process *P*_*i*_. The node *G* represents the spatial random effect, specified as a conditional autoregressive (CAR) model. The data object W is the associated adjacency matrix of the spatial lattice, while parameters *λ*_*G*_ and *τ*^2^ define the process’s overall spatial dependency and variance, respectively. *G* is shared between both the sampling effort components (dark blue block) and the ecological components (green block).

Our model assumes that records of observed organisms are determined by the joint effect of two types of random components: an ecological process *P*_*i*_ that determines the suitability of each taxon *i* (e.g. species) to settle in (or occupy) a given location, and a process *S* that identifies a preferential sampling that biases the records of observed occurrences. This process is assumed to be the same for all the taxa of interest. Regarding their general structure, both type of process share similar specifications. That is, a linear term (i.e. fixed effect) and a random effect with spatial autocorrelation. Despite their similarity, both processes represent distinct phenomena and, therefore, their likelihood and covariates are different. To give an overview of the model’s structure we present a directed acyclic graph (figure 1) representing the model’s conditional dependencies and its parameters. Hence, the model’s joint probability distribution can be factorized into the following product of independent terms:

2.1 $$\;\begin{array}{rl} & \left[y,Q,P,S,G,\alpha ,\beta_{p},\beta_{s},\tau^{2},\lambda_{G};d_{e},d_{a},W]=\prod_{i=1}^{n}[y_{i}|Q_{i}][y^{c}|S,G,\beta_{s};d_{a}\right]\\ & \;[Q_{i}|P_{i},S,\alpha_{i}][P_{i}|G,\beta_{\;p_{i}};d_{e_{i}}][S|G,\beta_{s};d_{a}]\\ & \;[G|\lambda_{G},\tau^{2};W][\beta_{\;p_{i}}][\beta_{s}][\lambda_{G}][\tau^{2}]. \end{array}$$

The left-hand term includes all the parameters of the model. To ease readability, symbols in bold on the left-hand side refer to the union of all taxa (i.e. *i* ∈ {1, …, *n*}). In this sense, **y** represents the observations of all (*n*) taxa of interest (*y*_*i*_ are the observations of a specific taxon *i*) plus the observations of the complementary sample (*y*^*c*^). That is, **y** = [*y*_1_, …, *y*_*n*_, *y*^*c*^]. Following this rule, the linear coefficients and environmental covariates are written as $\beta_{p}=[\beta_{\;p_{1}},\ldots ,\beta_{\;p_{n}}]$ and $d_{e}=[d_{e_{1}},\ldots ,d_{e_{n}}]$, respectively. The vectors *β*_*s*_ and *d*_*a*_ are the equivalent terms for the sampling effort process. Additionally, data-related variables are written in lower-case letters (e.g. *y*_*i*_ or *d*_*a*_), while latent variables are written in upper case (e.g. *P*_*i*_). Latent variables P and S correspond to the ecological and preferential sampling process, respectively. The combinations (also called mixture) of both processes are represented by the latent variables Q. The spatial random effect is represented by *G* and uses a spatial lattice for identification. The spatial lattice is a structure represented by a graph (i.e. a set of vertices and edges) composed of a finite number of non-overlapping areal units (also called *cells*) that cover a geographical region (i.e. a spatial lattice is a tessellation). Each area unit represents a spatial polygon and it is mapped one-to-one with a node in the graph. As such, any edge in the graph connects two area units. In the case of spatial lattices, the connection represents two neighbouring areas (i.e. areas that share a common border). Here, we denote the adjacency matrix of the aforementioned graph (i.e. the spatial lattice) with W. Additional parameters of the latent variables are shown in Greek letters. For a full description of the model refer to the electronic supplementary material. The design of the model assumes that the ecological and sampling effort processes generate random binary outcomes that are registered as presence or absence of taxa. As such, the corresponding specification of processes *P*_*i*_ and *S* is given by a logistic regression. Let us first describe the ecological process *P*_*i*_ as follows:

2.2 $$\text{logit}\;([P_{i}(x_{k})|G(x_{k}),\beta_{i};d_{e_{i}}])=\beta_{P_{i}}^{t}d_{e_{i}}(x_{k})+G(x_{k}),$$

where $\beta_{P_{i}}\in R^{r}$ is a vector of linear coefficients for the fixed effect, $d_{e_{i}}(x_{k})\in R^{r}$ the *r*-dimensional vector of environmentally related (i.e. scenopoetic) covariates, corresponding to location *x*_*k*_ and *G* the *spatial random effect* that describes the spatial autocorrelation.

The sampling effort (*S*) is modelled in a similar form

2.3 $$\text{logit}\;([S(x_{k})|G(x_{k}),\beta_{s};d_{a}])=\beta_{s}^{t}d_{a}(x_{k})+G(x_{k}).$$

In this case, $\beta_{s}\in R^{s}$ is a vector of linear coefficients for the fixed effect of the sample and $d_{a}(x_{k})\in R^{s}$ an *s*-dimensional vector of anthropological (or sample-related) covariates corresponding to location *x*_*k*_. While the likelihood of each *P*_*i*_ depends directly on the presence (and absence of evidence) of a specific taxon *i*, the likelihood of *S* could rely, as we will expose later, on aggregated observations from other *complementary* taxa. This has the purpose to provide an *informed guess* of a sampling process common to all taxa of interest.

The model specifies a convex combination of *P*_*i*_ and *S* to express the combined effect between the ecological process and the preferential sampling. This combined effect is denoted by *Q*_*i*_ and is formally defined as

2.4 $$[Q_{i}(x_{k})|P_{i}(x_{k}),S(x_{k}),\alpha_{i}]=\alpha_{i}P_{i}(x_{k})+(1-\alpha_{i})S(x_{k}),$$

where 0 ≤ *α*_*i*_ ≤ 1. This parameter can be interpreted as the proportion of variation attributable to the ecological process. Throughout this text, we refer to this parameter as the *contribution to ecological suitability* for a given taxon *i* (*i* ∈ {*i*, …, *n*}) in location $x_{k}\in W$.

### 2.1. Shared spatial effect

The spatial autocorrelation between observations is specified as a latent variable *G* shared between all taxa (*P*_*i*_), including the sampling process (*S*). The shared random effect in the model specification (i.e. *G*) captures the spatial variability by correlating observations that are within neighbouring locations. That is, locations that share a border (or a vertex) are correlated to each other (i.e. a correlation different from zero), while locations that do not share borders (or vertices) are assumed to be independent (i.e. correlation equal to zero). Here, the term location refers to areas bounded by a closed polygon and, therefore, the set of all locations in a given region is a finite set of areas. This set, together with the neighbour relationship defines a spatial lattice (denoted by the symbol W). The lattice structure is equivalent to a simple graph (using the areas as vertices and the neighbour relations as edges). The process that generates *G* over the spatial lattice structure is specified with a proper conditional autoregressive model (CAR) [46]. In this specification, the spatial lattice (through its adjacency matrix representation) serves as a correlation structure to define the covariance matrix of a zero-centred multivariate normal distribution (MVN). In this context, *G* is called a Gaussian–Markov random field [47].

The CAR specification restricts the applicability of the model to observations aggregated on each area element of the spatial lattice. As such, all observations contained in a given area are projected into a single value to represent presence, absence or missing information. This methodology eases the computational complexity during inference, given the sparse form of the adjacency-matrix. The resulting implementation uses fast and efficient numerical methods that reduce the processing time by several orders of magnitude. Additionally, the shared specification of *G* (i.e. *G* is shared with all *P*_*i*_ and *S*) allows the random effect to act as a source of information exchange between all the taxa, as the inference of each individual taxon affects the inference of the rest of the processes. For a full description of the model and its common spatial random effect refer to the electronic supplementary material.

### 2.2. Support for missing data

The model allows inference on locations where information about the presence or absence of a given taxa (or sampling effort) is unknown. This approach treats missing observations as additional parameters to the model. The approach is similar to the data augmentation scheme proposed by Tanner & Wong [48]. It uses the information provided by the latent variables (*Q*, *P*, *S* and *G*) to sample posterior distributions at the locations with missing information. The scheme is incorporated into the MCMC-based inference, along with the rest of the parameters. Aided by the spatial autocorrelation structure (*G*) and the data from nearby areas, the fitted posterior distribution can provide information about the presence (or absence) at sites with missing data. Refer to the electronic supplementary material for a formal specification of the posterior distribution on sites with missing data.

### 2.3. Using complementary taxa to fit the sampling effort

The identification of the model using presence-only data requires the specification of additional assumptions about absences [28]. In this model, we follow a similar approach to presence-background SDMs [37] by using the presence of other taxa (i.e. complementary to the taxa of interest) as informative background data. Its associated observations (referred as *y*^*c*^ in figure 1) are used to fit the sampling process *S*, conditional to the common spatial effect *G*. Here, we propose the use of the natural taxonomic classification of life (a classification based on the evolutionary relationships between organisms) to determine the set of background data. The informative set, hereafter called *complementary* sample (*y*^*c*^), serves as a basis to define pseudo-absences and fit the sampling effort process using a different set of covariates specific to the sampling effort (see equation (2.1)). In previous work, we demonstrated that the use of an external informative sample and a common random spatial effect increased the model’s predictive accuracy in single PO-SDM [49]. In this work, we generalized a similar approach for multispecies models.

We begin by defining a *universal* set of taxa (hereafter called Ω) as the basis for the analysis. The selection of Ω will depend on each application, as it could be restricted at convenience to fit a given region, a given group of taxa or during a certain period of time. Given that each taxon is a node of the tree of life (ToL), the set Ω induces a subtree (referred as $T_{\Omega }$) by inheriting the taxonomy of the ToL. In this setting, the taxa of interest (*N*_*y*_) is a subset of the nodes of $T_{\Omega }$. We denote the subtree of interest as $T_{N_{y}}$. The corresponding complementary tree of $T_{N_{y}}$ (denoted by $T_{N_{y}}^{c}$) is obtained by the difference between $T_{N_{y}}$ and the subtree of $T_{\Omega }$ generated by selecting the lowest common node of *N*_*y*_ (LCA(*N*_*y*_)) in $T_{\Omega }$. The nodes of the complementary tree (i.e. $T_{N_{y}}^{c}$) correspond to the complementary taxa. This set is denoted as $N_{y}^{c}$. A detailed explanation of the methodology used for obtaining $\Omega ,T_{\Omega },T_{N_{y}},T_{N_{y}}^{c}$ and $N_{y}^{c}$ as well as how to root the taxonomic tree ($T_{\Omega }$) is provided in the electronic supplementary material.

The presented methodology is motivated by the assumption that the disjoint set of closely related occurrences (in a phylogenetic or taxonomic sense) gives information about a sampling effort common to all the taxa of interest. The characterization of this common sampling effort would serve to reduce the bias in presence-only data by including taxonomically related taxa sampled in the same region. The observations of the complementary taxa $N_{y}^{c}$ support the likelihood of the sampling effort *S* with informative data derived entirely by the taxonomic classification. It is important to mention that this methodology for generating a complementary sample is not a requisite for implementing the proposed model. The practitioner could use any other type of informative data to fit the sampling effort process. Additionally, to avoid ambiguity, we assume a unique taxonomic tree of life in space and time.

#### 2.3.1. Obtaining the complementary sample

The complementary sample is defined as the binary response vector *y*^*c*^ ∈ {0, 1}^*k*^ that represents presence or absence of the complementary taxa over the lattice W (composed of *k* unit areas). The response *y*_*i*_ is defined in algorithm 1. An implementation for obtaining $T_{\Omega },T_{N_{y}}$, $T_{N_{y}}^{c},\mathrm{LCA}(N_{y})$ and $N_{y}^{c}$ was achieved with the software Biospytial [50], a graph-based knowledge engine for ecological data. A script for obtaining the taxonomic trees and complementary sample is included in the electronic supplementary material.


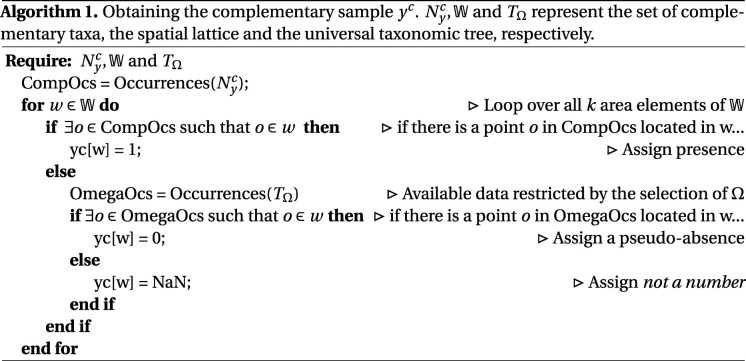


### 2.4. Model implementation

The model was implemented in STAN [51], a Turing complete probabilistic programming language for specifying statistical models. STAN performs full Bayesian inference using Markov chain Monte Carlo methods such as Hamiltonian Monte Carlo sampling. It also includes the $\hat{R}$ statistic [52] as a robust diagnostic for chain convergence. The implementation code is located in the electronic supplementary material.

## 3. Validation with simulated data

To validate the model implementation, we generated a synthetic dataset following the specification of the model. The model was fitted using a Hamiltonian Monte Carlo approach that resulted in posterior samples for each model’s parameters. All the parameters used for generating the synthetic dataset were inside the 95% credible interval of their corresponding fitted posterior sample. The complete specification, analysis, results of the simulation and work related to data acquisition are described in the electronic supplementary material.

## 4. Application

To illustrate the applicability of the model, we provide two case studies with different selections of the taxa of interest (ToI); a first case where the ToI are taxonomically distant and a second case where the selected taxa are closely related (i.e. all are from the same family).

### 4.1. Case study 1: occurrences of taxonomically diverse taxa

In this study, the selection of the taxa of interest was based on the following criteria: (i) Each taxon should be abundant and distributed widely across the region. (ii) The selected taxa should respond to known environmental factors, in particular elevation and precipitation. (iii) Documented mutualistic relationships between the taxa exist. (iv) Taxonomic diversity of the taxa should be preferred to ensure a diverse complementary sample. The main motivation for these criteria was to assess the model’s capabilities to produce realistic results based on abundant and well-studied taxa. In addition, to find a balance between these criteria, the taxa were constrained to family or genus.

Based on an exploratory analysis, we selected a set of abundant genera and families that satisfied the aforementioned criteria. This set was composed of: leadtrees (genus: *Leucaena*, family: Fabaceae), oaks (genus: *Quercus*, family: Fagales), pines (family: Pinacea), leafnose bats (family: Phyllostomidae) and woodpeckers (family: Picidae). The three groups of plants respond to gradients of elevation and precipitation, while the two selected animals respond to ecological relationships with the associated biomes where the chosen plants are abundant. All the taxa have implicit ecological relevance as they shelter and support the existence of other species.

Leadtrees are shrubs associated with tropical semi-deciduous forests and seasonal lowland forest. They can live between the 0 m to 1400 m.a.s.l. [53]. Oaks comprise trees and shrubs. They are frequently distributed between 1200 to 2800 m.a.s.l. and between 600 to 1200 mm of precipitation per year. They are commonly associated with pines in *mixed* forests. However, it is also common to find them in mesophyl forests, grasslands and woodlands [54]. Pines prefer to live between temperate to cold dry climates, moderately moist and acid soils. Apart from being associated with oaks in *mixed* forests, they are also found with cypresses and spruces. Pines have affinity to live between 1500 to 3600 m.a.s.l. and tolerate a wide range of precipitation conditions. Depending on the species this restriction can range from 350 mm to 1000 m [54]. leafnose bats constitute the most diverse family of bats (Order: Chiroptera) and include frugivorous, insectivorous and haematophagous. The family Phyllostomidae, in particular, is a taxon of high ecological importance, as they provide key ecosystem functions like pollination, seed dispersal, nutrient cycling and arthropod suppression [55,56]. Woodpeckers are the most abundant group of birds. The selection of this group was due to its strong association with woodland forests, in particular with oaks and pines.

#### 4.1.1. Explanatory variables

The covariates used as explanatory variables for the ecological process were elevation and annual mean precipitation. The elevation data were obtained from the Global Relief Model *ETOPO1* at 1 arc-minute resolution [57]. The precipitation data were obtained from the World Climatic Data *WorldClim* version 2 [58]. These data are distributed as a 12 band raster model with *ca* 1 km spatial resolution averaged across months to produce a mean temperature value per pixel. The anthropological covariates, used to explain the sample process, were distance to the closest road and population density. The distance to the closest road was generated in two steps. First, we rasterized the National Road Network for Mexico (*Red Nacional de Caminos* (RNC) INEGI, Instituto Mexicano del Transporte and Gobierno de Mexico [59], scale: 1:250 000) at 1 km spatial resolution. Later, we used this raster dataset to calculate its proximity to the closest road (pixels flagged as road) using the function gdal_proximity [60]. The road network data were obtained from Vázquez [61]. The population dataset was obtained from the WorldPop project [62] for the year 2010. The dataset consists of population counts on each areal element, each with a spatial resolution of 3 arc-seconds (*ca* 100 m). All the explanatory variables were spatially overlaid and aggregated by mean on each areal element.

#### 4.1.2. Study region

**Figure 2.**
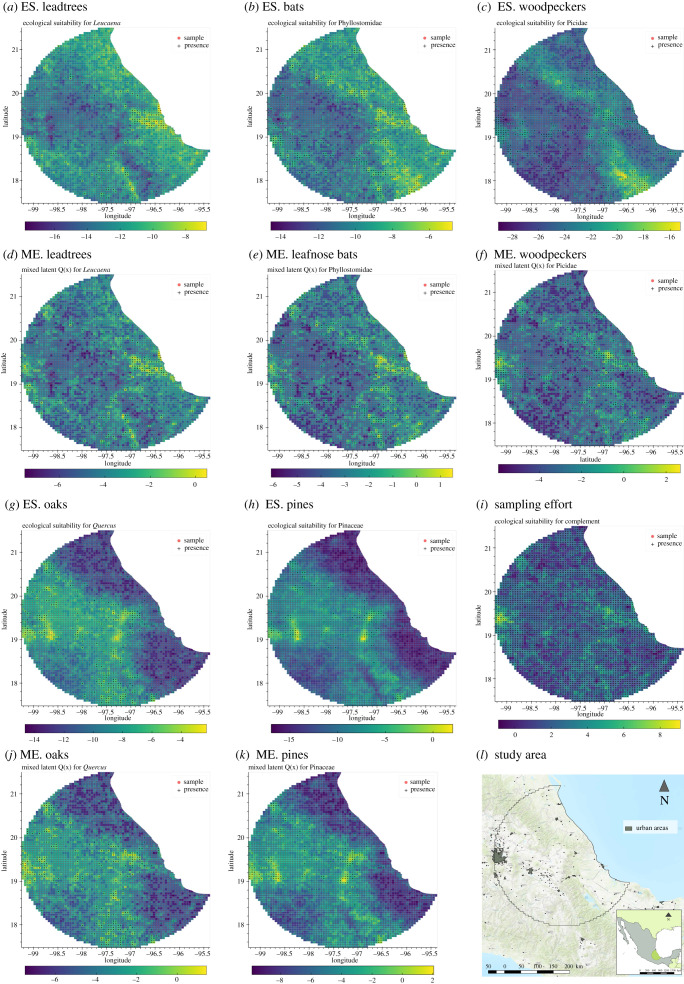
Comparison of ecological suitability (ES.) processes *P*_*i*_ (*a*,*b*,*c*,*h*,*g*) and the corresponding mixing effect (ME.) *Q*_*i*_ (*d*,*e*,*f*,*j*,*k*) in the study area (*l*). *Q*_*i*_ is the convex combination of *P*_*i*_ with the sampling effort process *S* (*i*). All figures show the respective mean posterior on each unit element of the lattice W in the study area (*l*).

The study area covers approximately 112 000 km^2^ and intersects several Mexican states (e.g. Veracruz, Puebla, Hidalgo, Mexico City and Oaxaca). It includes heterogeneous landscapes with variability in geomorphological and climatic features as well as distinct biomes such as coastal dunes, chaparrales, mesophyl forests, evergreen rain forest, grasslands, mangroves, broad-leaf forests and coniferous forests [54,63]. Figure 2*l* shows the region over Mexico. The region under study is of ecological importance due to the confluence of the two biogeographic realms in the American continent; neartic and neotropical [64]. Consequently, the region is rich in biodiversity at several taxonomic levels and, therefore, of high scientific interest. Additionally, the collection of GBIF records is highly abundant facilitating the acquisition of informative complementary samples.

#### 4.1.3. Geographic lattice W

The lattice W used was obtained from a polygon intersected on a geographical grid of approximately 4 km spatial resolution. The region comprises the inland area of a circular polygon centred at 19 N–97 E with radius of 2° (*ca* 200 km). The area covers approximately 112 000 km^2^ and is composed of 4061 spatial units (see figure 2*l* in electronic supplementary materials I). To derive the associated adjacency matrix *W* we performed a topological analysis on the grid to determine the corresponding neighbours for each areal unit. To ease the processing work on data acquisition and transformation to the adjacency matrix representation, we used *Biospytial* [65].

#### 4.1.4. Occurrence and taxonomic data

The biodiversity data used were all the available GBIF occurrences [31] registered before January 2015, constrained to the studied region (4.1.3). The raw data were downloaded from GBIF (doi:10.15468/dl.oflvla). For further information on this dataset, including data attributions, see GBIF.org [66].

The GBIF dataset was cleaned to procure consistent querying of taxonomic trees and the correct execution of graph-based and geospatial algorithms. The cleansing procedure included: removal of records with incomplete taxonomic information, removal of records with incomplete geospatial coordinates and removal of records with incomplete acquisition dates. As such, each GBIF record includes full taxonomic and spatio-temporal information. The occurrences contained inside each area were parsed into a taxonomic-tree structure using the taxonomic classification of the GBIF Taxonomic Backbone [67]. Therefore, for the 4061 area elements in the lattice (W) we obtained the same number of taxonomic trees (hereafter referred to local taxonomic trees). To obtain the *complementary sample* (§2.4), all the local taxonomic trees were merged into a single regional tree (i.e. T*(*N*_*y*_)). The complementary sample was generated by applying algorithm 1.

To obtain the vector of observations (*Y*_*i*_) for each taxon of interest *i* a *point in polygon* test was performed. That is: *Y*_*i*_(*x*) = 1 if taxon *i* is present in areal element *x*, otherwise *Y*_*i*_(*x*) = 0. In this case study, we assume no missing information on the sample effort to reduce to a minimum the amount of sample-dependent parameters introduced by the data augmentation methodology. Sample-dependent parameters increase the model uncertainty. Assessing the model performance subject to these treatments is out of the scope of this particular case study. The data processing pipeline, as well as the generation of local and regional taxonomic trees was also undertaken with *Biospytial*.

#### 4.1.5. Model fitting

The response vectors, together with their respective covariates, were arranged in a design matrix with shape (4061 * 6) × (2 * 2), where 4061 is the number of areal elements of the lattice W and 6 corresponds to the five taxa plus the sample. The 2 * 2 columns correspond to two columns for the ecological covariates and two columns for the anthropological covariates.

For fitting the model, we used our implementation in the STAN language (see electronic supplementary material). We obtained posterior samples through MCMC using the NUTS sampler on four independent chains with default parameters of step size and tree depth. The posterior sample was run for 35 000 iterations with a burn-in size of 17 500 and thinning of 35. The prior distributions for *β*_*i*∈{1, … ,*n*}_ are distributed *N*(0, 10 000). The prior distribution for parameters *α*_*i*_ (mixing process *Q*_*i*_) and *λ*_*G*_ (proper CAR model) are β(5, 5) and the parameter *τ* is distributed as Inv. Γ(1, 0.01).

#### 4.1.6. Results

The posterior means and credible intervals of the model’s parameters are shown in table 1. In all the MCMC chains (4), all parameters converged ($\hat{R}<1.1$) according to the $\hat{R}$ diagnostic for convergence [68]. Analysis among taxa showed that leadtrees (*Leucaena*) obtained significant negative correlation with elevation and precipitation while leafnose bats (Phyllostomidae) showed positive correlation for precipitation and negative correlation for elevation. Oaks (*Quercus*) and pines (Pinacea) showed significant preference for higher elevations below the tree line. However, pines also showed significant preference for precipitation. Woodpeckers (Picidae) showed preference for higher precipitation but no significant preference for elevation.

**Table 1.** Posterior means, 95% credible intervals and convergence diagnostic $\hat{R}$ for the first case study using taxonomic diverse taxa in the eastern part of Mexico. Ecological suitability and sampling effort corresponds to the processes *P* and *S* defined in the main text. The row with the contribution to ecological suitability describes the parameter *α*_*i*_ defined in the mixing process *Q*_*i*_, for each taxon *i*.

				credible intervals				
			mean	2.5%	50%	97.5%	n.eff	$\hat{R}$
ecological suitability (*P*)	intercept	*Leucaena*	−9.06	−15.39	−8.64	−5.23	725	1.0
		Phyllostomidae	−10.13	−16.42	−9.67	−6.39	900	1.01
		Picidae	−30.35	−57.26	−28.07	−15.59	218	1.02
		*Quercus*	−11.84	−17.67	−11.46	−8.16	1176	1.0
		Pinacea	−18.0	−26.15	−17.48	−12.82	1172	1.0
	elevation	*Leucaena*	−1.7 × 10^−3^	−2.9 × 10^−3^	−1.6 × 10^−3^	−9.3 × 10^−4^	1185	1.0
		Phyllostomidae	−8.0 × 10^−4^	−1.4 × 10^−3^	−7.7 × 10^−4^	−3.6 × 10^−4^	1675	1.0
		Picidae	4.9 × 10^−4^	−3.8 × 10^−4^	4.5 × 10^−4^	1.6 × 10^−3^	1068	1.0
		*Quercus*	1.9 × 10^−3^	1.3 × 10^−3^	1.9 × 10^−3^	2.8 × 10^−3^	1453	1.0
		Pinacea	3.5 × 10^−3^	2.5 × 10^−3^	3.5 × 10^−3^	5.1 × 10^−3^	1194	1.0
	precipitation	*Leucaena*	−0.01	−0.03	−0.01	−4.0 × 10^−3^	2770	1.0
		Phyllostomidae	9.8 × 10^−3^	3.1 × 10^−3^	9.5 × 10^−3^	0.02	1487	1.0
		Picidae	0.04	0.02	0.03	0.07	243	1.01
		*Quercus*	−3.6 × 10^−3^	−0.01	−3.5 × 10^−3^	3.8 × 10^−3^	4234	1.0
		Pinacea	0.03	0.02	0.03	0.04	1375	1.0
	sampling effort (S)	intercept	2.56	2.37	2.56	2.76	309	1.02
		distance to road	−1.5 × 10^−4^	−1.9 × 10^−4^	−1.5 × 10^−4^	−1.1 × 10^−4^	976	1.01
		population density	4.3 × 10^−4^	3.2 × 10^−4^	4.3 × 10^−4^	5.5 × 10^−4^	858	1.01
	contribution to ecological suitability (*α*)	*Leucaena*	0.44	0.28	0.44	0.62	866	1.0
		Phyllostomidae	0.44	0.29	0.44	0.6	1031	1.01
		Picidae	0.2	0.1	0.2	0.33	331	1.01
		*Quercus*	0.51	0.36	0.51	0.65	1350	1.0
		Pinacea	0.58	0.42	0.59	0.74	1520	1.0
	spatial effect	*λ*_*g*_	0.13	0.05	0.12	0.23	1149	1.0
		*τ*	0.1	0.09	0.1	0.12	113.0	1.03

The sampling effort was found to be significant for both covariates (i.e. distance to closest road and population density) with an increasing probability for getting samples in places close to roads (negative correlation) and with high population density (positive correlation).

In relation to the ES associated with each taxon, we found that pines obtained the largest contribution with respect to the sampling effort (mean 0.58 with 0.42, 0.78 at 95% CI). This was followed by oaks (mean 0.51 with 0.35, 0.65 at 95% CI). Leadtrees and bats obtained similar results (mean 0.44, 0.28,0.62 at 95% CI) while woodpeckers obtained the smallest value (mean 0.2, 0.1, 0.3 at 95% CI).

The posterior means for the spatial effect showed low spatial dependence (*λ*_*G*_) of 0.13 ranged from 0.05 to 0.23 at 95% CI with an overall variance (*τ*^−1^) of 10 ranged from 0.833 to 11.11 at 95% CI.

To show the model’s capability to discriminate between the sampling effort process (*S*) and the ES (*P*_*i*_) of each taxon, we compared side-by-side the spatial process *P*_*i*_ with its corresponding mixed process (*Q*_*i*_, figure 2). It is remarkable that all the ES processes show smoother (less noisy patterns) than their corresponding mixed processes. Additionally, the probability of occurrences in urban areas, specifically the metropolitan area of Mexico City (see largest grey polygon in figure 2*l*) are attenuated in the ecological process. This effect is different for each taxa and is discussed in §4.2. Leadtrees, bats and woodpeckers are mostly distributed on the eastern side of the mountain ridge (Sierra Madre Oriental) while oaks and pines overlap and are distributed in higher areas of the mountain ridges.

The *k*-fold cross-validation resulted in high predictive accuracy with an AUC of 0.916 (0.914 −0.916). The used method and the resulting ROC curves are shown in the electronic supplementary material.

### 4.2. Case study 2: predicting cactii in Mexico

This case study aims to predict the underlying ES of four genera of cactii using the rest of the family Cactacea as complementary taxa. A key distinction with the former case study is the use of missing data (observations) in the sampling effort. In this case, missing data are defined in places where neither the presence of the taxa of interest nor the complementary sample exist. That is, there is no known record of any occurrence and, consequently, no arguable proof of absence of the taxa or an associated sample.

Cactii comprise all members of the family Cactacea. This group is likely to be a monophyletic, that is, all known species share a single common ancestor [69]. The evolutionary lineage of cactii suggests commonality in their traits and adaptations to the environment. However, there are some members of this group that, despite their limited tolerance to humid environments, have evolved to develop strategies for living in tropical ecosystems with intense precipitation. An example of this is the genus *Disocactus* that developed an epiphyte habit and, therefore, gathers water and other nutrients from the air.

In this case study, we analyse the capability of the model to discriminate the ES of the taxa distributed between two distinct ecosystems: shrublands–deserts (SD) and tropical rain forests (TRF). The group representing the SD ecosystems is composed of the genera: *Mammilaria*, *Coryphantha* and *Echinocactus*, the group corresponding to TRFs is represented by the genera *Disocactus*.

The habitat of the three chosen genera in the SD group overlaps significantly [70]. However, the data availability of the three genera differs significantly, partly due to the sampling effort and the ecological rareness of each genus. This study also demonstrates the capacity of the model to improve the inference of rare species given the information of more common taxa.

#### 4.2.1. Study region

This case study covers all Mexico, approximately 3200 km in length over an area of 1 972 550 km^2^. According to the Köppen classification, the country has 15 different climate types. The variety of these climates, its large geographical extent and complex geomorphology distinguish the country as a megadiverse region, hosting two biogeographic realms: Neartic and Neotropical.

#### 4.2.2. Explanatory variables

The explanatory variables (i.e. covariates) used in the ecological process were: annual mean temperature, annual mean precipitation and elevation. As in the first case study, these variables were obtained from the World Climatic Data *WorldClim* version 2 [58] and the Global Relief Model *ETOPO1* [57]. Compared with the first case study, the wider geographical extent and coarser spatial resolution required the use of different covariates to fit the sampling effort process. Recalling that these covariates should represent general differences between surveys, we opted to use two sources of land cover classification: the Terrestrial Ecoregions of the World [71] and Conabio’s typology of human settlements by municipality (*Tipología municipal por asentamiento humano, CONABIO* [72]). As these two datasets are categorical, we transformed each category into a binary column (dummy variable). The resulting number of covariates for both categorical datasets was 18.

#### 4.2.3. Geographic lattice

**Figure 3.**
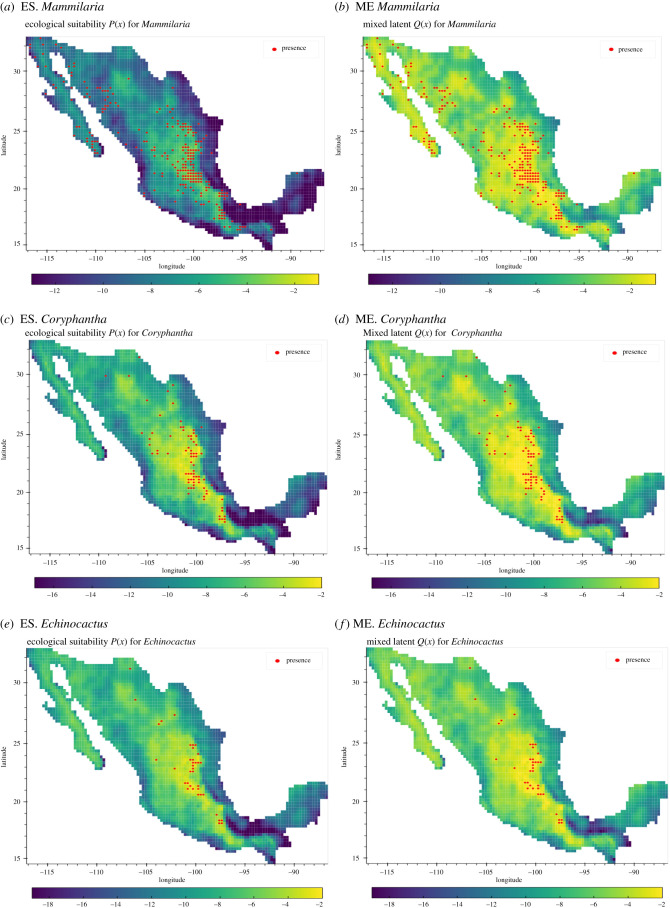

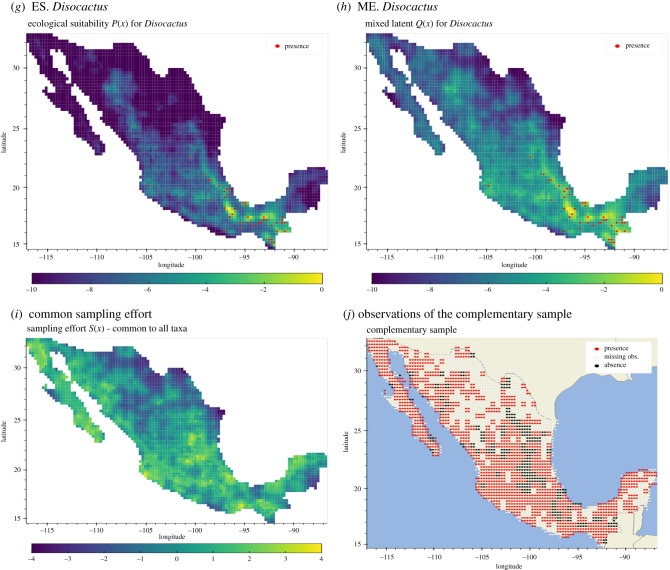
Comparison of the posterior mean ecological suitability (ES.) *P*_*i*_ and the mixed effect (ME.) between ES and a common sampling effort for *Mammilaria* (*a*,*b*), *Coryphantha* (*c*,*d*), *Echinocactus* (*e*,*f*) and *Disocactus* (*g*,*h*). The last two panels show the posterior mean of the common sampling effort (CSE) process *S* and the observations of the complementary sample used for fitting the CSE.

The lattice used in this study was obtained by overlapping the *Marco Geoestadístico de Mexico* [73] with a regular rectangular grid with approximately 20 km resolution. The resulting spatial lattice is composed of 2941 cells and only considers the continental portion of the country (i.e. all islands have been excluded). The observations of the sampling effort were aggregated to a coarser resolution of 40 km^2^ approx. The resulting composition of areas consisted of: 1659 presences (56%), 356 pseudo-absences (12%) and 926 missing observations (31%). For a graphical description, see figure 3*j*.

#### 4.2.4. Occurrence data

Similarly to case study 1 (§4.1), the data used for occurrences were the complete set of GBIF occurrences [31] registered before January 2015 contained in continental Mexico. The raw data were downloaded from GBIF (DOI:10.15468/dl.oflvla) and have the same metadata as in case study 1. For further information on this dataset, including data attributions, see GBIF.org [66]. The occurrences contained inside each area were parsed into a taxonomic-tree structure using the taxonomic classification of the GBIF Taxonomic Backbone [67]. The complementary sample was generated by algorithm 1 upon previous selection of the taxa of interest and their LCA (in this case, the taxonomic node Cactacea). The process for obtaining observations was the same as in case study 1. However, a key difference in this study is the definition of missing observations in places where no information of both the complementary sample and the observations was obtained (see algorithm 1). This methodology allows inference on more realistic assumptions about the absence of observations at the expense of increasing the model complexity and the overall uncertainty. The data processing pipeline, as well as the generation of local and regional taxonomic trees, was also undertaken with *Biospytial*.

#### 4.2.5. Model fitting

The response vectors, together with their respective covariates, were arranged in a design matrix similar to the specifications of case study 1 (§4.1) this time with shape (2941 * 5) × (3 * 2), where 2941 is the number of areal elements of the lattice W and 5 corresponds to the four taxa plus the sample. The 3 * 2 columns correspond to three columns for the ecological covariates and two columns for the anthropological covariates.

For fitting the model we used our implementation in the STAN language (see electronic supplementary material). We obtained posterior samples through MCMC using the NUTS sampler on four independent chains with default parameters of step size and tree depth. The posterior sample was run for 35 000 iterations with a burn-in size of 17 500 and thinning of 35. The data were normalized previously and the prior distributions of the parameters *β*_*i*∈{1, … ,*n*}_ were distributed *N*(0, 100). For the parameters *α*_*i*_ (mixing process *Q*_*i*_) and *λ*_*G*_ (proper CAR model), the prior distribution were beta(5, 5). The parameter *τ* had a prior distribution of Inv. Γ(1, 0.01).

#### 4.2.6. Results

The posterior means and credible intervals of the fitted parameters are shown in table 2. The $\hat{R}$ diagnostic for convergence [68] showed that all parameters in all the MCMC chains (4) converged.

**Table 2.** Posterior means, 95% credible intervals and convergence diagnostic $\hat{R}$ for the second case study (cactii) in continental Mexico. Ecological suitability and sampling effort correspond to the processes *P* and *S* defined in the main text. The row with contribution to ecological suitability describes the parameter *α*_*i*_ defined in the mixing process *Q*_*i*_, for each taxon *i*. Taxa in bold indicate a significant effect (95% CI) of the respective variable. In the case of the sampling effort, only the significant variables are shown.

				Credible intervals				
			mean	2.5%	50%	97.5%	n.eff	$\hat{R}$
ecological suitability (*P*)	elevation	*Disocactus*	0.81	−0.38	0.79	2.08	4627	1.0
		***Mammilaria***	2.41	1.59	2.39	3.31	1716	1.0
		***Coryphantha***	3.41	2.46	3.4	4.38	1156.0	1.0
		***Echinocactus***	3.19	2.11	3.19	4.3	1275	1.0
	precipitation	***Disocactus***	1.8	1.39	1.8	2.24	509	1.0
		***Mammilaria***	−2.08	−2.81	−2.08	−1.37	2184	1.0
		***Coryphantha***	−2.8	−3.77	−2.78	−1.88	1198	1.0
		***Echinocactus***	−3.1	−4.31	−3.08	−1.98	1414	1.0
	mean temperature	*Disocactus*	−0.33	−1.52	−0.35	0.88	4735	1.0
		*Mammilaria*	0.63	−0.24	0.63	1.53	4012	1.0
		***Coryphantha***	1.24	0.29	1.24	2.21	4208	1.0
		***Echinocactus***	1.64	0.47	1.64	2.78	3715	1.0
	sampling effort (*S*)	intercept	6.57	4.77	6.59	8.33	828	1.0
		forests woodlands and scrubs	2.25	0.48	2.24	4.0	4103	1.0
		dry broadleaf forests	1.38	0.09	1.37	2.72	4223	1.0
		metropolitan	2.09	0.82	2.09	3.37	3846	1.0
		rural	1.71	0.5	1.7	2.95	4043	1.0
		urban	1.72	0.49	1.71	3.0	4073	1.0
	contribution to ecological suitability (*α*)	*Disocactus*	0.71	0.62	0.71	0.8	282	1.0
		*Mammilaria*	0.41	0.36	0.41	0.47	241	1.0
		*Coryphantha*	0.68	0.58	0.68	0.77	309	1.0
		*Echinocactus*	0.78	0.67	0.78	0.87	425	1.0
	spatial effect	*λ*_*g*_	0.99	0.99	0.99	0.99	4031	1.0
		*τ*	0.46	0.36	0.45	0.58	1675	1.0

Overall, in terms of the ES of the taxa, the model showed consistent results with their natural history. Precipitation was found to be significant in all selected taxa. Moreover, a positive correlation for *Disocactus* was found, a species reported to inhabit tropical rain forests (TRF), and negative correlation for all the genera associated with shurblands and deserts (SD). Elevation showed significant positive correlation in the ES of the taxa associated with shrublands and deserts (SD). Slightly different was the case of the mean annual temperature; although a variable commonly correlated with elevation, it showed to be non-significant for the presence of *Disocactus* or *Mammilaria*.

In the case of the sampling effort, and particularly among the terrestrial ecoregions, only the dry broadleaf forests and forests woodlands and scrubs were found to be significant for characterizing the sampling effort. By contrast, the majority of typologies for human settlements were significant (i.e. metropolitan, urban and rural areas).

In relation to the contribution to the ES process (parameter: *α*_*i*_), we found that for *Echinocactus*, *Disocactus* and *Coryphantha* a high proportion of their variation was attributable to the environmental niche (i.e. ES), while the presence of *Coryphantha* was heavily dependent on the overall sampling effort (*α* = 0.41).

In the case of the spatial random effect, the posterior means for parameters associated with this effect showed a maximal spatial dependence (*λ*_*G*_ = 0.99 ± (0.995, 0.998) at 95% CI) and the overall variance (*τ*^−1^) was found to be moderate 0.46 ± (0.36, 0.58) at 95% CI.

A side-by-side comparison between the ES (*P*_*i*_) of each taxon (*i*) and the corresponding mixed process (*Q*_*i*_) is shown in figure 3. The spatial distribution, obtained from the posterior sample of the ES process, is consistent with the ecology of the four taxa. That is, *Mammilaria*, *Coryphantha* and *Echinocactus*, have a common spatial distribution consistent with the distribution of deserts and shrublands (Central and Northern Mexico). As expected, the distribution of *Disocactus* is different from the others. While the taxa associated with the group SD are prevalent in the central-west and northern regions of the country (arid shrublands and deserts), *Disocactus* presents the highest prevalence in South Mexico, a region with high precipitation and presence of tropical rain forests.

## 5. Discussion

The analyses of both case studies suggest that the variance of the model (driven by the parameters *τ*^2^ and *λ*_*g*_) is a determining factor for the precision of the model (range of the credible intervals) and the greater the values of the parameters, the harder it is to identify accurately the spatial effect. Although the results presented correspond to a single simulation, they indicate the precision that may be expected for real data.

The findings in case study 1 showed that leadtrees (*Leucaena*) have negative preference for elevation and precipitation. This result is consistent with ecological theory as it is a plant that thrives in warm and semi-arid environments [53,54]. Interestingly, pines and oaks obtained similar estimates for their contribution to their respective ES (and sampling effort). These two taxa are often considered a single type of vegetation due to their complex network of dependencies and similar ecological niches [54]. The fact that both taxa have similar contributions as well as an overlapping geographical space (i.e. middle to high elevated areas of the Sierra Madre Oriental) is indicative of their common ecological niche and suggests that the presence of one taxon is informative of the other.

Leafnose bats have a preference to roost in warm subtropical regions [74]. Consistent with ecological theory, areas with higher ES for bats were the subtropical regions between the coast and the foothills of the Sierra Madre Oriental (figure 2*b*). Moreover, constrained to the study area, the highest levels of ES coincide remarkably with the neotropical realm, suggesting that the model is capable of capturing macroecological patterns.

The fixed effect for the common sampling effort was significant for both covariates (i.e. distance to closest road and population density) supporting the idea that these two covariates are important for characterizing the sampling process.

The low contribution to ES for Leadtrees, bats and woodpeckers, in particular, suggests that the observations of these taxa are mostly biased by the sampling process. This is consistent with the fact that birds and mammals are among the most overrepresented taxa in biodiversity occurrence data [75]. Leadtrees have many agricultural and industrial uses such as: shade for coffee plantations, forage for cattle and their seeds and resin are used for fabricating perfumes and soaps [53]. Their extensive use explains the strong contribution of the sampling effort, mostly driven by anthropological covariates.

In the second study case, the model demonstrated its capacity to infer locations with missing observations. Consistent with their ecology, the taxa associated with SD ecosystems present similar spatial distributions with varying degrees of intensity, arguably dependent of the number of observations of each taxa. By contrast, *Disocactus* presents a distinctive spatial distribution. In this case, the regions with higher intensity are located in the southern regions of the country, predominantly to the west of the Tehuantepec Isthmus and the state of Chiapas (figure 3*g*). Consistent with the ecology of the genus, the regions with high-intensity overlap with tropical rain forests.

### 5.1. Reduction of the sampling effort bias

The contribution to the ES of each taxon *i* (i.e. parameter *α*_*i*_) can be interpreted as the proportion of variation attributable to the ecological process given the overall process *Q*_*i*_. Alternatively, 1 − *α*_*i*_ can related to the proportion of variation attributable to the sampling effort. This parameter allows the identification of two processes: one determined by the specific environmental niche of each taxon and another, driven by the commonality dictated by an abstract sampling effort. In both case studies, the parameter *α* showed to be a useful measure of the sampling bias. Additionally, *α* allowed the identification of processes *P* and *S*, reducing the sampling bias in the ecological niche, and providing more information on the underlying sampling effort. For example, an interesting result was the attenuation of the ES process (*P*) in large urban areas. When compared with the mixed process (*Q*), *P* showed reduced probability in those areas, despite the overrepresented observations in areas close to metropolitan areas.

The attenuation effect suggests that the signal of scientific interest (i.e. the ES) can be recovered from observations affected by the mixed effect between the ecological and sampling signals. The signal recovery varies depending on the taxon. For example, woodpeckers (figure 2*c*) were less likely to occur in the metropolitan area of Mexico City (middle western part of the study area), once the signal from the sampling process was removed (see figure 2*f* for comparison). This result is consistent with the reported preference of woodpeckers and other forest birds to nest in natural and semi-urban areas rather than urban and densely populated areas [76].

In the case of cactii, the mixed processes *Q* showed higher probability values when compared against their respective *P* (figure 3) in the same location. That is, given a fixed location *x* the estimated probability of *Q*_*i*_(*x*) was higher than *P*_*i*_(*x*) for taxon *i*. This phenomenon was present in all taxa. However, the effect was notable in *Disocactus*, where the intensity across the arid regions in the northern part of Mexico (*P_Disocactus_*) was greatly reduced when compared with its mixed process *Q_Disocactus_* (figure 3*g* and 3*h*, respectively). Another interesting case is the genus *Mammilaria*, in which the ecological process (figure 3*a*) was attenuated in regions with high precipitation, despite the relative higher proportion of occurrences and consequently, higher intensity surface of *Q_Mammilaria_* (figure 3*b*).

### 5.2. Computational limitations and scalability

The model presented here is a type of hierarchical multilevel model. As such, its scalability is limited by the computational complexity of the model and its implementation. In case study 2 (cactii), the numbers of parameters to be estimated were: 2943 spatial parameters (areal elements of the lattice), 10 coefficients of mixed proportions (*α*), 356 missing observations, 15 anthropological and three environmental covariates; giving a total of 3327 parameters. This specification was implemented in a computer with a quad-core Xeon 9 CPU and 9 GB of RAM. The posterior sample consisted of 35 000 iterations on four independent chains and took 12.5 h to complete.

Scaling the model to *k* more taxa will increment the number of parameters to *k* × *n*; where *n* is the number of areal elements of the lattice (in case study 2 *n* = 2943). Additionally, a specification in which the number of missing data is larger than the total number of observations (presences and pseudo-absences) would induce problems of parametric identification and divergences and, therefore, we recommend to perform an exploratory analysis prior to the implementation of the approach.

An alternative for specifying a more parsimonious spatial random effect could be the use of a geostatistical approach (e.g. Gaussian process) that reduces the number of spatial parameters to three or four (e.g. Diggle *et al.* [77]). Although this approach is sensible, the implementation could yield a much higher computational complexity due to the operations involved in dense matrix inversion against sparse matrix factorization defined by lattice-based models [78]. We welcome new contributions that could reduce the complexity of the presented model.

### 5.3. Roads for future research

The use of missing data in case study 2 allowed the examination of the model’s predictive capacity in areas with missing observations, that is, areas with no known presences nor pseudo–absences of the complementary sample. The results show that, in sites with missing observations (figure 3*j*), the predicted underlying intensity surface was lower (dark regions in figure 3*i*) than in sites with presence or pseudo-absences. Although these regions present higher uncertainty, given by the data augmentation methodology, they represent an interesting phenomenon worth to be explored in the future. Additionally, the study alone of the sampling effort process gives an idea of the state of the sample given an arbitrary set of taxa, the role of the anthropological factors affecting the distribution of the overall sampling effort and its spatial correlation. We think that exploring this model’s feature could aid in detecting gaps in biodiversity collections as well as prioritizing study sites.

There are other potential routes for future research. A strong assumption of the presented model is that the spatial random effect (*G*) is shared between all the taxa of interest and the sampling effort. While this model is able to capture spatial interactions between taxa, we think that these interactions could be more ecologically meaningful if the spatial effect is modelled jointly, accounting for correlated spatial components between the sampling process and one (or multiple) ecological suitability(ies). A way to move forward in this direction is to specify a multivariate CAR (MCAR) model [79] for the spatial effect (*G*) to account for joint and marginal spatial effects for each *P*_*i*_ and *S*.

The complementary sample approach may not be appropriate for all types of taxa of interest due to the high complexity of phylogenetic, phenotypic and environmental relationships, specifically in the role of interspecific competition and facilitation (see [80] review and [81]). As such, we suggest caution when applying the complementary sample algorithm on any group of taxa without analysing previously possible relations of spatial exclusion due to potential competition. Increasing the taxonomic resolution to subranks (e.g. subfamilies, tribes, subgenera) can be a first step in obtaining more informative complementary samples. However, an improved algorithm aimed at integrating phylogenetic relationships in theoretically consistent assemblages of ecological communities should account for both spatial and phylogenetic effects, of overdispersion and clustering (see [82] for a conceptual definition and [83] for a semantic methodology).

## Data accessibility

The library is available in: https://github.com/molgor/JSDM4POD/. It includes the implemented Python and STAN models, working examples on both case studies, data (compressed) and interactive Jupyter notebooks [84]. We intend to put the code and data in a long term curated repository such as Dryad or FigShare. The data are provided in the electronic supplementary material [85]. The model is available as an Open Source (GPL) Python library called JSDM4POD (i.e. a joint species distribution model for presence-only data).
